# Head-to-head comparison of pulsed-field ablation, high-power short-duration ablation, cryoballoon and conventional radiofrequency ablation by MRI-based ablation lesion assessment

**DOI:** 10.1007/s10840-025-02086-9

**Published:** 2025-06-24

**Authors:** Mariona Regany-Closa, Josep Pomes-Perez, Eric Invers-Rubio, Roger Borras, Berta Pellicer-Sendra, Susanna Prat-Gonzalez, Rosario Jesus Perea, Carlos Igor Morr, Jean-Baptiste Guichard, Elena Arbelo, Jose Maria Tolosana, Eduard Guasch, Andreu Porta-Sanchez, Marta Sitges, Josep Brugada, Ivo Roca-Luque, Lluís Mont, Till F. Althoff

**Affiliations:** 1 Department of Cardiology, Hospital Clínic Cardiovascular Institute (ICCV), Universitat de Barcelona, C/Villarroel N° 170, 08036 Barcelona, Catalonia Spain; 2 Institut d’Investigacions Biomèdiques August Pi I Sunyer (IDIBAPS), C/del Rosselló, 149, 08036 Barcelona, Catalonia Spain; 3 Centro de Investigación Biomédica en Red Cardiovascular (CIBERCV), Av. Monforte de Lemos, 3-5, 28029 Madrid, Spain; 4 Department of Cardiology, Angiology and Intensive Care Medicine, German Heart Center of the Charité (DHZC), Charitéplatz 1, 10117 Berlin, Germany

**Keywords:** Atrial fibrillation, Pulsed-field ablation, High-power short-duration ablation, Cryoballoon ablation, Cardiac magnetic resonance, Ablation lesions

## Abstract

**Background:**

Novel concepts for pulmonary vein isolation (PVI) like pulsed-field ablation (PFA) or high-power short-duration ablation (HPSD) promise favorable profiles of safety and efficacy. However, clinical comparisons of those novel concepts with conventional ablation approaches regarding ablation lesions are lacking. To systematically investigate lesion characteristics of novel ablation concepts, we performed a prospective head-to-head comparison using late gadolinium enhancement (LGE)-CMR.

**Methods:**

This study included patients undergoing first-time PVI-only atrial fibrillation ablation—either by ablation index–guided radiofrequency ablation (RF), cryoballoon ablation (CRYO), HPSD (90W, 4 s), or PFA (Farapulse). All patients received an LGE-CMR 3 months post-ablation to assess ablation lesions.

**Results:**

Post-ablation LGE-CMRs from 138 patients were analyzed (43 RF, 40 CRYO, 25 PFA, 30 HPSD). PFA resulted in the least continuous LGE lesion with the lowest proportion of complete PV-encircling LGE lesions (PFA 12%, HPSD 40%, RF 26%, CRYO 24%; *p* = 0.0069). Ablation with the CRYO and PFA single-shot devices resulted in the widest lesions. Of note, HPSD lesions were significantly wider than conventional RF lesions (PFA 12.7 mm, HPSD 10.9 mm, RF 8.7 mm; CRYO 13.3 mm; *p* < 0.0001).

**Conclusions:**

Lesion characteristics differed significantly among the four ablation techniques. HPSD ablation resulted in the most continuous LGE lesions. Of note, HPSD lesions were also wider than conventional RF lesions, thus corroborating the concept of a shallower HPSD lesion geometry from experimental studies. PFA lesions cover relatively large areas but are more inhomogeneous. Wether this indicates ineffective ablation or lower detectability of PFA lesions by LGE-CMR remains unknown.

**Graphical Abstract:**

Ablation lesion characteristics differ significantly between ablation techniques, with high-power short-duration RF ablation (HPSD) resulting in the most continuous late gadolinium enhancement lesions in MRI.

**Figure:**
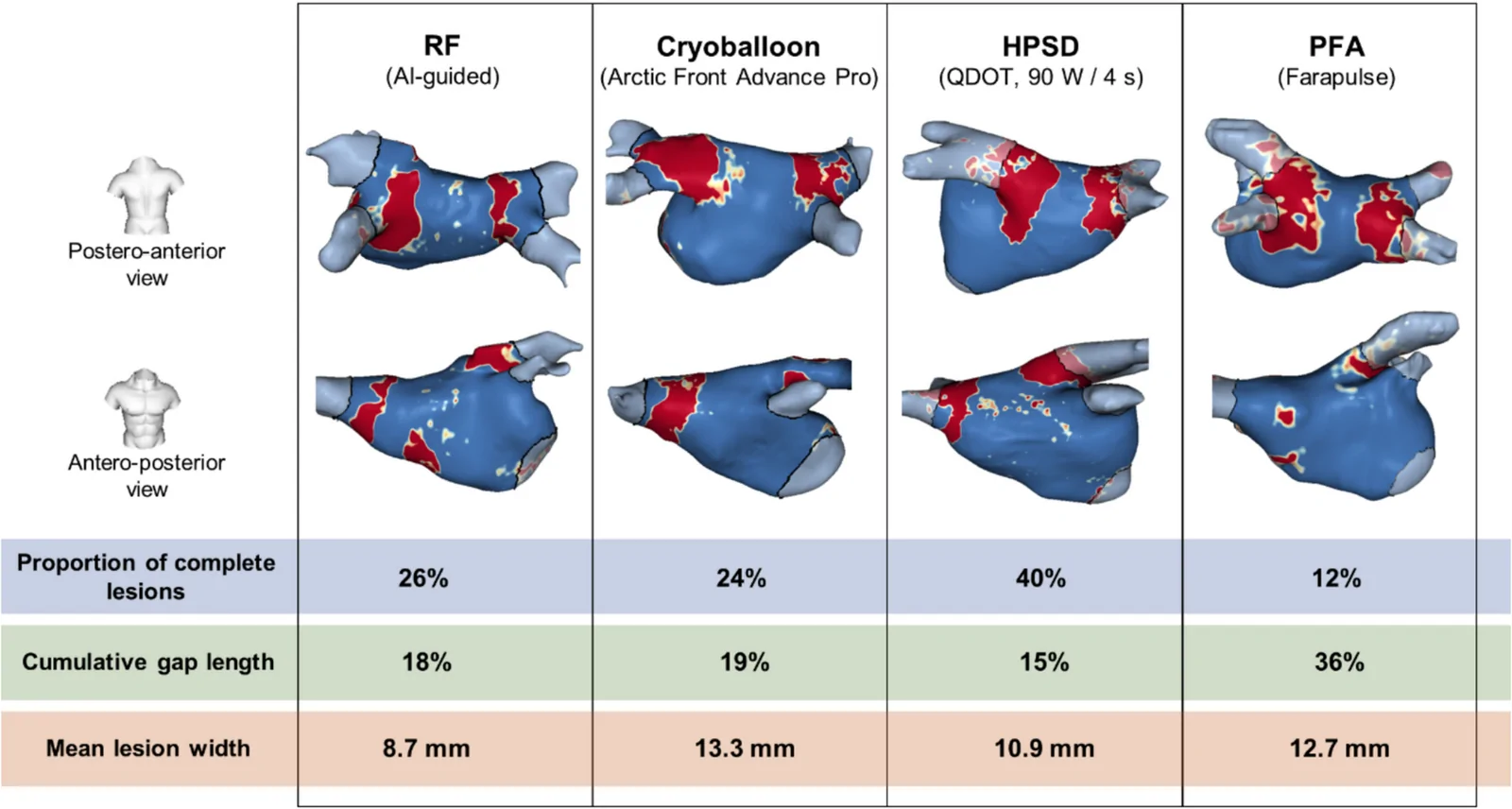

## Introduction

Pulmonary vein (PV) isolation (PVI) through catheter ablation is considered the most effective atrial fibrillation (AF) therapy [1]. PVI was consistently shown not only to reduce the risk of arrhythmia recurrence and AF burden, but also to prevent disease progression from paroxysmal to persistent AF [2].

However, despite substantial technological and procedural advances that have improved the efficacy and safety of AF ablation in recent years, PVI durability and long-term recurrence rates of more than 50%, even in patients with paroxysmal AF, are still not satisfactory [3, 4].

Of note, recent data suggest that there may be ablation technology–dependent differences in ablation lesion properties other than durability depending on the ablation technology, which result in differential effects on progression from paroxysmal to persistent AF [3]. Moreover, novel concepts for pulmonary vein isolation (PVI) like pulsed-field ablation (PFA) [5–13] or high-power short-duration radiofrequency ablation (HPSD) [14–19] promise improved safety and efficacy profiles due to cardiomyocyte selectivity and a favorable lesion geometry, respectively [20, 21]. However, a definite clinical proof of those benefits and a comparison with established concepts regarding lesion geometry and durability in clinical trials are lacking.

In recent years, late gadolinium enhancement (LGE) cardiac magnetic resonance (CMR) has been established as a non-invasive method to assess ablation lesions [22–30]. In fact, LGE-CMR reliably detects ablation-induced fibrosis, localizes gaps with high accuracy, and has been used to confirm durable PVI [22, 31, 32]. As a direct measure of ablation efficacy, LGE-CMR-based lesion assessment constitutes a valid efficacy endpoint, even with limited sample sizes [33, 34].

Against this background, to systematically investigate lesion characteristics of novel ablation concepts, we performed a prospective head-to-head comparison between PFA (Farapulse), HPSD ablation (QDOT, Biosense Webster), cryoballoon (CRYO) ablation (Arctic Front Advance Pro, Medtronic), and conventional ablation index–guided radiofrequency (RF) ablation (SmartTouch, Biosense Webster) using LGE-CMR.

## Methods

### Study design and participants

This was a prospective study including consecutive patients undergoing first-time, PVI-only PFA (Optiwave, Farapulse), HPSD ablation (QMODEplus, QDOTmicro catheter, Biosense Webster), CRYO (Arctic Front Advance Pro, Medtronic), or conventional RF ablation (ablation index–guided, Thermocool SmartTouch, Biosense Webster). All patients received an LGE-CMR 3 months after ablation and a systematic follow-up with outpatient visits including ECG and holter-ECG at 3, 6, and 12 months post-ablation. The study protocol was reviewed and approved by the local research ethics committee, and written informed consent was obtained from each patient.

### LGE-CMR image acquisition

LGE-CMR was performed as previously described [31, 32, 35]. In brief, CMR studies were performed in sinus rhythm using a 3-Tesla scanner (Magnetom Prisma, Siemens Healthineers, Erlangen, Germany) with 32-channel phase array cardiovascular coil.

Inversion recovery–prepared T1-weighted gradient echo sequences were acquired in axial orientation using ECG gating and a free-breathing 3D navigator, 20 min after administering an intravenous bolus of 0.2 mmol/kg of gadobutrol (Gadovist, Bayer Hispania).

#### Sequence parameters

Repetition time 2.3 ms, echo time 1.4 ms, flip angle 11°, bandwidth 460 Hz/pixel, and inversion time (TI) 280–380 ms, acquired voxel size 1.25 × 1.25 × 2.5 mm.

If image quality of the post-ablation LGE-CMR was deemed insufficient to allow for valid assessment of the study endpoints by the experienced, blinded investigator in charge, the respective patient was excluded from the analysis. Reasons that prohibited valid assessment of the study endpoints included motion artifacts, low signal-to-noise ratio, and suboptimal contrast enhancement.

### LGE-CMR post-processing

A novel artificial intelligence (AI)-based automatic algorithm embedded in a dedicated software (ADAS 3D, Adas3D Medical SL, Barcelona, Spain) was employed for left atrial 3D segmentation as well as quantification of LGE lesions and gaps. This algorithm uses a well-established machine learning approach consisting of a localizer and a segmenter. Both, localizer and segmenter, apply distinct 3D variations of the widely used network architecture U-Net, a fully convolutional neural network developed for biomedical image segmentation at the Computer Science Department of the University of Freiburg, Germany [36, 37].

The automatic algorithm was systematically validated using as a reference two highly experienced experts (M.R., J.P.), blinded with respect to the study groups. For 3D reconstruction of left atria and PVs, operators traced the atrial wall on each axial-plane slice, which was then automatically adjusted to build a 3D shell.

**Fig. 1:**
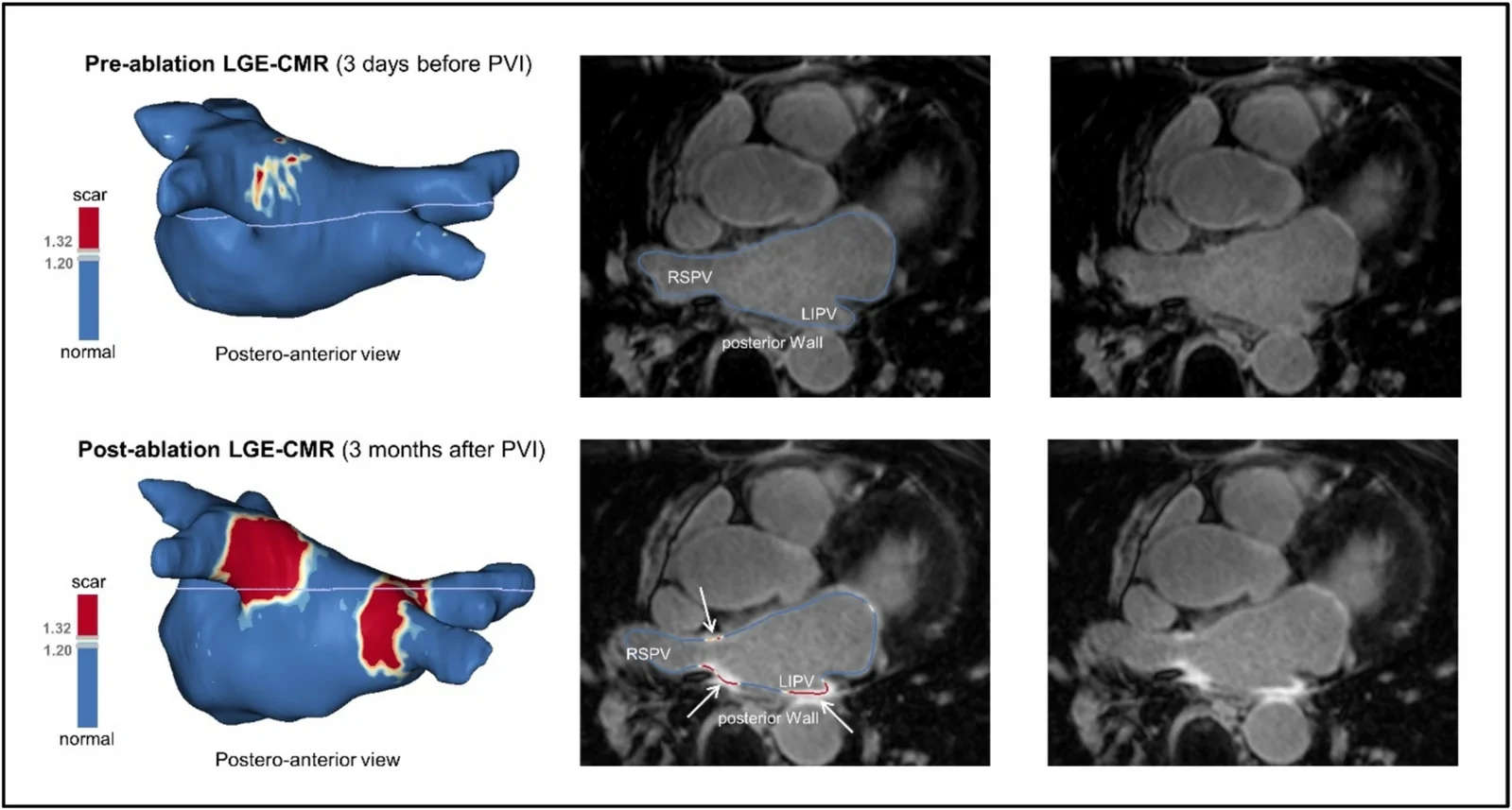
LGE-CMR-based detection and visualization of ablation lesions after PVI. Left: 3D reconstruction of the left atrium with color-coding based on signal intensity ratios with distinct thresholds for scar (red, > 1.32; yellow, 1.2–1.32) and normal tissue (blue, < 1.2) (ADAS 3D). Blue lines indicate the plane of the left atrial slices on the right. Middle: Overlay of the T1-weighted images with the LGE color-coding described above. White arrows point to local ablation-induced LGE lesions. Right: T1-weighted LGE-CMR slice depicting the LA with evident LGE of PV antral walls. RSPV, right superior pulmonary vein; LIPV, left inferior pulmonary vein)

LGE was quantified in a standardized manner based on signal intensities of each given voxel relative to the mean blood pool signal intensity, applying a previously validated signal intensity ratio threshold (SIR) of ≥ 1.2 to define LGE indicative of fibrotic tissue [31, 32]. Automatic 3D-reconstructions, which were color-coded accordingly, were created using the ADAS-3D software (Fig. 1).

### Quantification of ablation lesions and gaps

#### Number of gaps

LGE lesions around the PV ostia were defined based on the aforementioned signal intensity ratio threshold (≥ 1.2). Any LGE discontinuation of ≥ 3 mm was considered a gap (previous studies from our group suggest that LGE discontinuations of < 3 mm may not be relevant for clinical outcome and that consideration only of LGE discontinuations ≥ 3 mm does not significantly lower the sensitivity in the detection of gaps) [31, 32].

#### Cumulative gap length

The cumulative length of all gaps in the PV-encircling LGE lesion was determined and related to the full peri-PV antral circumference (the perimeter of the anticipated ablation line). Thus, cumulative gap length was expressed in percent, relative to the peri-PV antral circumference. Based on this parameter, a dichotomous variable was defined applying a cutoff of 10% cumulative gap length. This variable is based on previous evidence indicating that neither the number of gaps nor the size of single gaps, but rather the cumulative gap length relative to the total perimeter of the assumed ablation line, is predictive of clinical outcome [38, 39]. In fact, it has been demonstrated that a cumulative gap length of ≥ 10% is associated with substantially elevated recurrence rates.

#### Ablation lesion geometry

To compare ablation lesion geometries, the width of the circumferential LGE lesions was measured analogous to an annular ring width. The maximum lesion width was determined for each of eight predefined peri-ostial PV segments perpendicular to the anticipated ablation line, and the mean of these maximum segmental widths was calculated (mean lesion width).

### Ablation procedure

Pulmonary vein isolation was performed under general anesthesia. Intravenous heparin was administered and titrated to ensure an activated clotting time of ≥ 300 s upon transseptal access.

#### PFA (Farapulse)

For the PFA procedure, a single transseptal access was established at the operator’s discretion—either by direct puncture with a steerable sheath (Faradrive, Farapulse, Boston Scientific, Marlborough, MA, USA), or with a non-steerable sheath (Swartz SL1, Abbott, Chicago, IL, USA) which was subsequently exchanged over the wire for the steerable sheath.

Guided by fluoroscopy, a five-spline PFA catheter (Farawave, 31-mm radius, Farapulse, Boston Scientific, Marlborough, MA, USA) in flower configuration was positioned at the PV ostium over the wire and then transformed into a basket configuration.

PFA was applied according to the Optiwave protocol as described previously [8]. After two PFA applications in basket configuation, the splines were rotated by 30–40° followed by two additional PFA applications to achieve overlapping lesions. Subsequently, four analogue applications were performed in the more antral flower configuration. Additional applications were performed at the operator’s discretions until proof of PV entrance block. This procedure was repeated for all PVs.

#### HPSD ablation

Single transseptal access was established with a steerable sheath (Vizigo, Biosense Webster, Johnson & Johnson, New Brunswick, NJ, USA). Subsequently, contact force calibration of the irrigated ablation catheter (QDOT micro, Biosense Webster) and respiration gating were accomplished, followed by fast anatomical mapping (CARTO 3 System, Biosense Webster Inc., Johnson & Johnson, New Brunswick, NJ, USA). If available, a 3D CT- or CMR-derived left atrial geometry was merged into the navigation system. Point-by-point RF ablation encircling ipsilateral pulmonary veins was performed in QMODE + (90 W, 4 s). The target intertag distance was 3.0 mm at the anterior wall, 4.0 mm at the posterior wall, and 5 mm in the proximity of the oesophagus. PVI was confirmed by entrance- and exit block using the micro-electrode ablation catheter. The maximum tolerable intertag distance was 6.0 mm. In the absence of first-pass PVI (i.e., no isolation upon completion of the encirclement of ipsilateral vein ostia), additional touch-up applications using QMODE (ablation index–guided ablation using 40–50 W) were performed at the operator’s discretion until PVI was accomplished.

#### Ablation index–guided RF ablation

Dual transseptal access was established for an ablation catheter (ThermoCool SmartTouch, Biosense Webster, Johnson & Johnson, New Brunswick, NJ, USA) and a multipolar mapping catheter (PentaRay, Biosense Webster, Johnson & Johnson, New Brunswick, NJ, USA). A non-steerable sheath (SL1, Abbott Medical Devices, or Preface, Biosense Webster, Johnson & Johnson, New Brunswick, NJ, USA) was used for the mapping catheter, whereas the use of a steerable sheath for the ablation catheter (Agilis, Abbott Medical Devices or Vizigo, Biosense Webster) was at the operator’s discretion. Subsequently, contact force calibration of the irrigated ablation catheter and respiration gating were accomplished, followed by fast anatomical mapping (CARTO 3 System, Biosense Webster Inc., Johnson & Johnson, New Brunswick, NJ, USA) of the left atrium using the multipolar mapping catheter. Ablation index–guided point-by-point RF ablation encircling ipsilateral pulmonary veins was performed in power control mode (SmartAblate, BiosenseWebster, Inc.) with 40 W at the anterior wall and 30 W at the posterior wall. Ablation index targets were 450 anteriorly and 350 posteriorly. Ablation was stopped when the esophageal probe indicated a significant temperature rise. While intertag distances between 3.0 and 5.0 mm were targeted, the maximum tolerable intertag distance was 6.0 mm. In the case of dislocation of the ablation catheter or failure to accomplish ablation index or intertag distance targets, additional RF applications were applied until targets were reached. PVI was confirmed by entrance and exit block using the multipolar mapping catheter. In the absence of first-pass PVI (i.e., no isolation upon completion of the encirclement of ipsilateral veins), PVI was accomplished with additional RF touch-up applications at the investigator’s discretion [40].

#### Cryoballoon ablation

For the CRYO procedure, a single transseptal puncture was performed with a non-steerable sheath (Swartz SL1, Abbott Medical Devices, or Preface, Biosense Webster) which was subsequently exchanged over the wire for a steerable sheath (FlexCath, Medtronic, Minneapolis, MN, USA). All procedures were performed using the 28-mm cryoballoon (Arctic Front Advance Pro, Dublin, Ireland). The PV potentials were recorded using the spiral PV catheter (Achieve Advance catheter, Medtronic, Dublin, Ireland). Selective angiography was performed for each PV to assess ostial anatomy and the degree of occlusion.

The minimum duration of cryo-applications was 180 s per PV. Applications that failed to isolate the PV within 60 s or to achieve a temperature of ≤ − 40 °C within 60 s were considered ineffective and terminated (thereafter the cryoballoon was re-positioned and a new application delivered). If real-time PV potential monitoring was not feasible, cryo-ablation was applied for 240 s. Additional applications were performed as needed until proof of entrance and exit block. Temperatures below − 55 °C prompted termination of the application. A diagnostic catheter was placed at the superior vena cava to pace and capture the phrenic nerve during ablation of the right PVs. Phrenic nerve capture was continuously assessed by the operator (tactile feedback).

Adaptations of the ablation strategy to anatomical variations like, e.g., a left common PV trunk were at the investigator’s discretion.

### Statistics

Continuous variables are presented as the mean value ± standard deviation. To compare the means of two variables, we used the Student’s *t* test and Mann–Whitney *U* test as appropriate. Categorical variables were expressed as total number (percentages) and compared between groups using chi-square test. Lin’s concordance correlation coefficient was used to assess the agreement between the the automatic algorithm for LA segmentation and gap detection and a blinded operator. We analyzed repeated measures using generalized multilevel mixed effect models where for all models, participant identification was treated as a random effect to model the within-participant correlations. To perform the *p*-value adjustment for multiple comparisons, Tukey strategy was used. To fit those models, we used Lme4 R package (v.1.1–35.3). Residual plots were used to perform model validation. Statistical comparisons and marginal means were computed with emmeans library (v. 1.10.1). For all tests, a *p*-value < 0.05 was considered significant. Statistical analysis was performed using R software for Windows 11 version 4.3.2 (R project for statistical computing; Vienna, Austria).

## Results

A total of 158 consecutive patients with first-time PVI-only AF ablation and post-ablation LGE-CMR were included. Twenty patients (13%) had to be excluded because of insufficient LGE-CMR quality. Thus, 138 patients were analyzed. Of those, 43 patients had undergone ablation index–guided RF ablation, 40 patients CRYO ablation, 30 patients PFA, and 25 HPSD ablation.

**Fig. 2:**
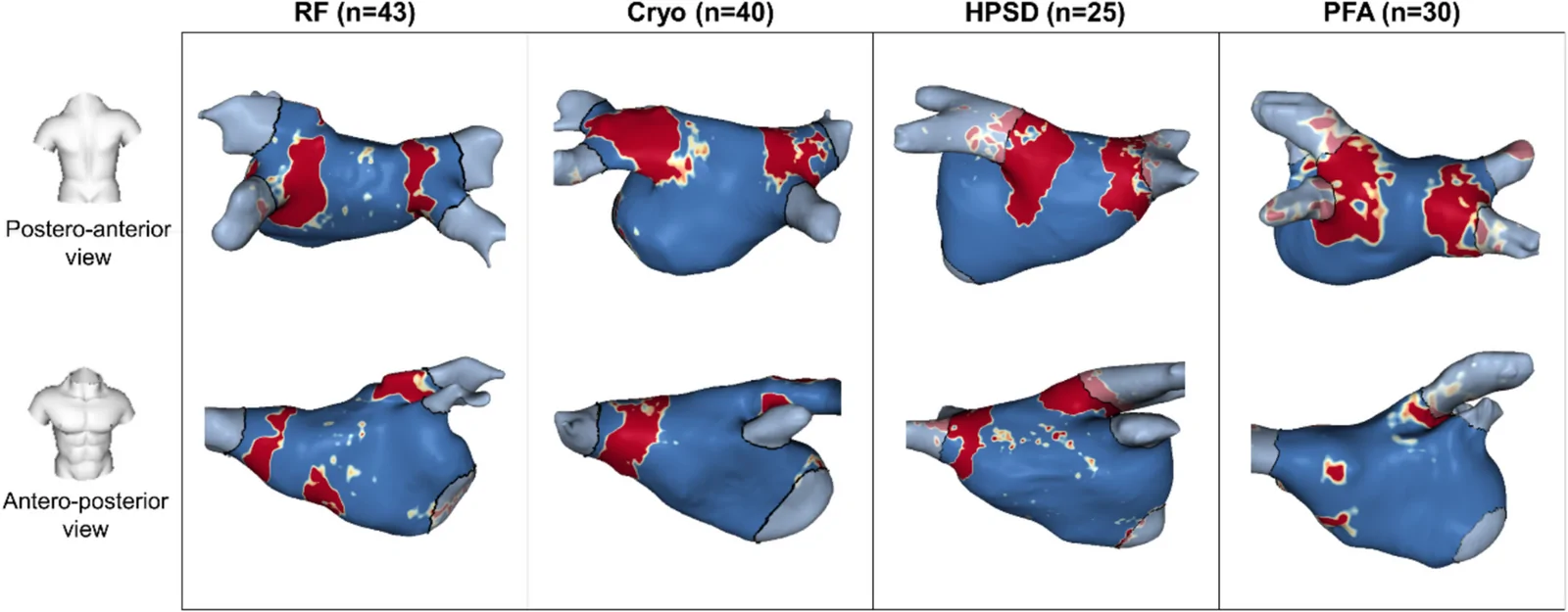
Representative LGE lesions sets according to ablation approach (central illustration). 3D reconstructions of left atria 3 months post-ablation (PVI-only) with color-coding based on signal intensity ratios with distinct thresholds for scar (red, > 1.32; yellow, 1.2–1.32) and normal tissue (blue, < 1.2) (ADAS 3D)

Complete PVI was confirmed in all patients during the ablation procedure. Representative 3D-reconstructions of LGE-CMR at 3 months post-ablation are displayed in Fig. 2. Patient characteristics are displayed in Table 1.

**Table 1:** Patient characteristics

	Overall *n* = 138	RF *n* = 43	CRYO *n* = 40	HPSD *n* = 25	PFA *n* = 30	***p***-value
Age (years)	58.5 (10.2)	60.1 (9.5)	52.9 (11.1)	62.7 (6.2)	60.4 (9.9)	< 0.001
Female sex	33 (24)	9 (21)	14 (35)	2 (8)	8 (28)	0.087
CHA_2_DS_2_VASc score	1.3 (1.2)	1.4 (1.3)	0.9 (1.1)	1.9 (1.1)	1.4 (1.1)	0.002
Hypertension	64 (47)	20 (47)	12 (30)	16 (64)	16 (55)	0.040
Diabetes mellitus	13 (9.5)	4 (9.3)	2 (5)	6 (24)	1 (3.4)	0.061
LA diameter (mm)	40.7 (5.9)	43.0 (6.5)	39.1 (4.3)	39.4 (6.1)	40.5 (5.6)	0.006
LVEF (%)	58.2 (6.0)	58.0 (5.2)	58.9 (5.4)	56.1 (7.4)	59.3 (6.3)	0.291
AF type						< 0.001
paroxysmal	91 (66)	21 (49)	32 (80)	12 (48)	26 (87)	
persistent	47 (34)	22 (51)	8 (20)	13 (52)	4 (13)	

All values are *n*, (%), or mean ± standard deviation (median and interquartile if not normally distributed). *LA*, left atrium; *LVEF*, left ventricular ejection fraction

### Validation of AI-based automatic algorithm

In a first step, the automatic algorithm for 3D reconstruction and quantification of atrial volumes, LGE, and gaps was validated using the results from two experienced, blinded investigators as a reference. This validation demonstrated excellent inter-operator reliability as indicated by the following Lin’s concordance correlation coefficients [41]. Regarding quantification of left atrial volumes and LGE indicative of ablation lesions, agreement between the automatic algorithm and the blinded operators was excellent (0.94 and 0.95, respectively). Similarly, the agreement in the quantification of cumulative gap lengths and the proportion of patients with complete lesions was excellent (0.96), whereas agreement was somewhat lower but still substantial with respect to the absolute number of gaps (0.81).

### Lesion continuity

**Fig. 3:**
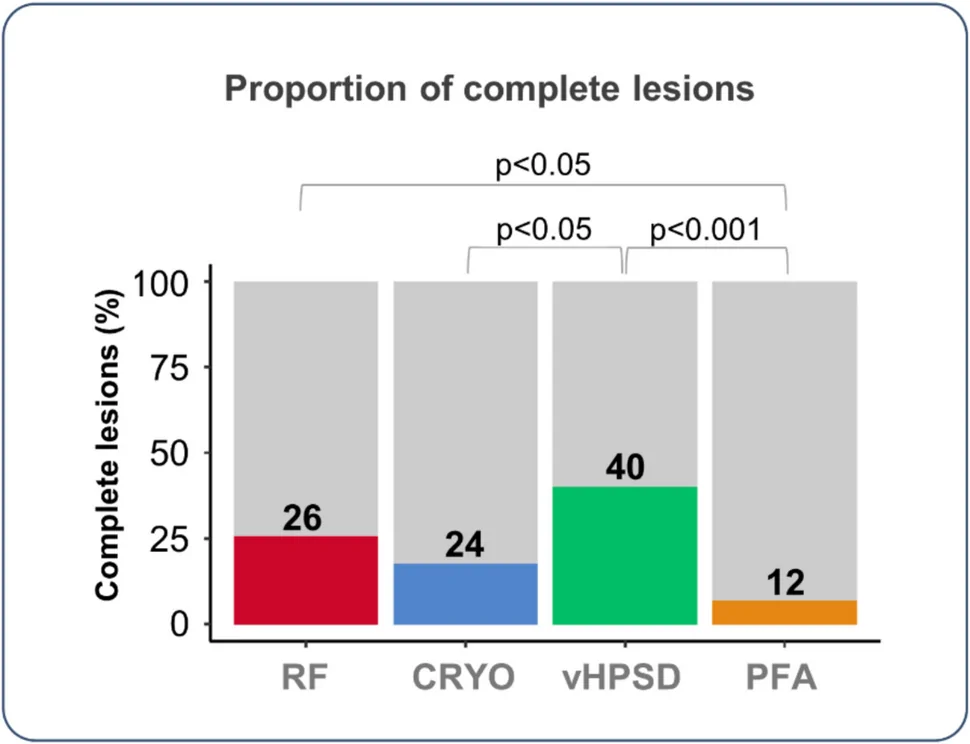
Complete lesions. Proportion of PV-encircling lesions without any gap (bold numbers signify means). Only *p*-values < 0.05 are displayed

**Fig. 4:**
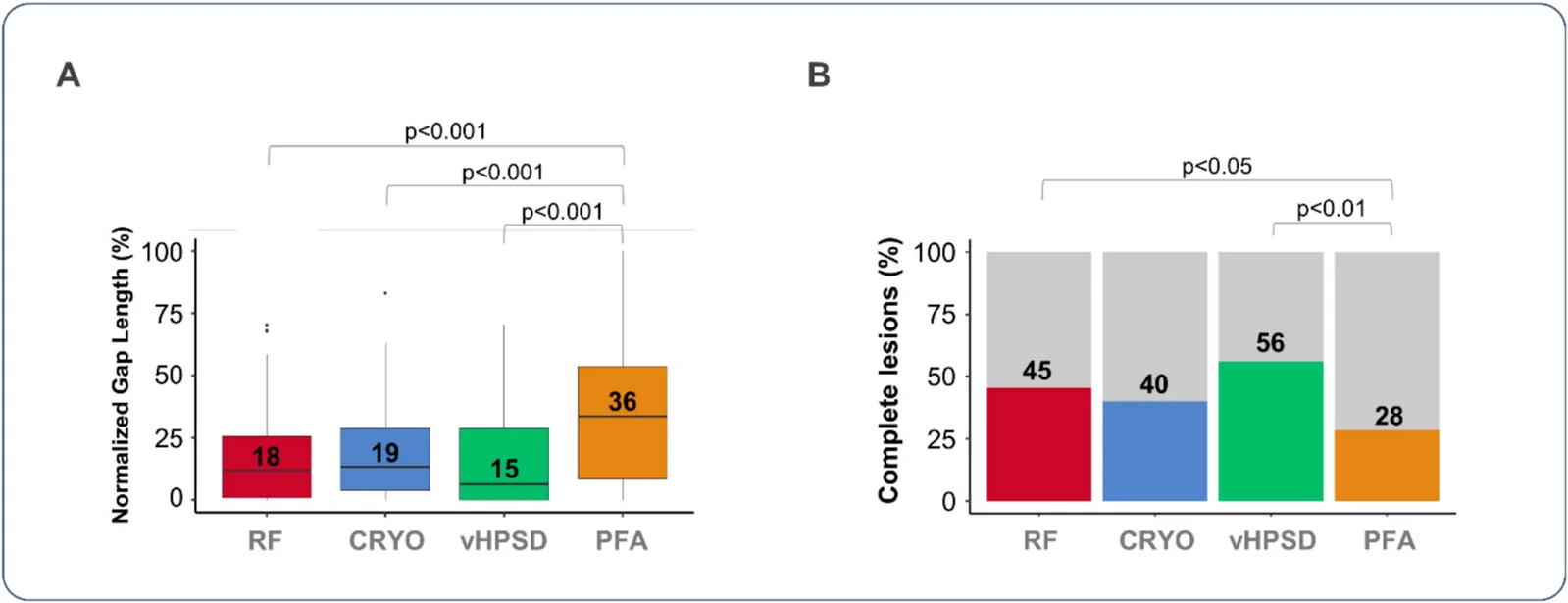
Cumulative gap length. **A** Cumulative gap length: The cumulative length of all gaps in a given PV-encircling LGE lesion relative to the full peri-PV circumference. **B** Proportion of PV-encircling LGE lesions with less than 10% relative gap length. Bold numbers signify means. Only *p*-values < 0.05 are displayed

Lesion continuity differed significantly between the different ablation techniques. The proportion of PV-pairs with continuous PV encircling lesions without any gap was 40% after HPSD, 26% after conventional RF, 24% after CRYO, and 12% after PFA (*p* = 0.0069; Fig. 3). In line with these data, the cumulative gap length was lowest with HPSD (15%) followed by ablation index–guided RF (18%), CRYO (19%) and PFA (36%) (*p* < 0.0001; Fig. 4A).

PV encircling LGE lesions covering > 90% of the antral perimeter (< 10% cumulative gap length) were encountered in 56% with HPSD ablation, 45% with conventional RF, 40% with CRYO ablation, and 28% with PFA (*p* = 0.0471; Fig. 4B).

**Fig. 5:**
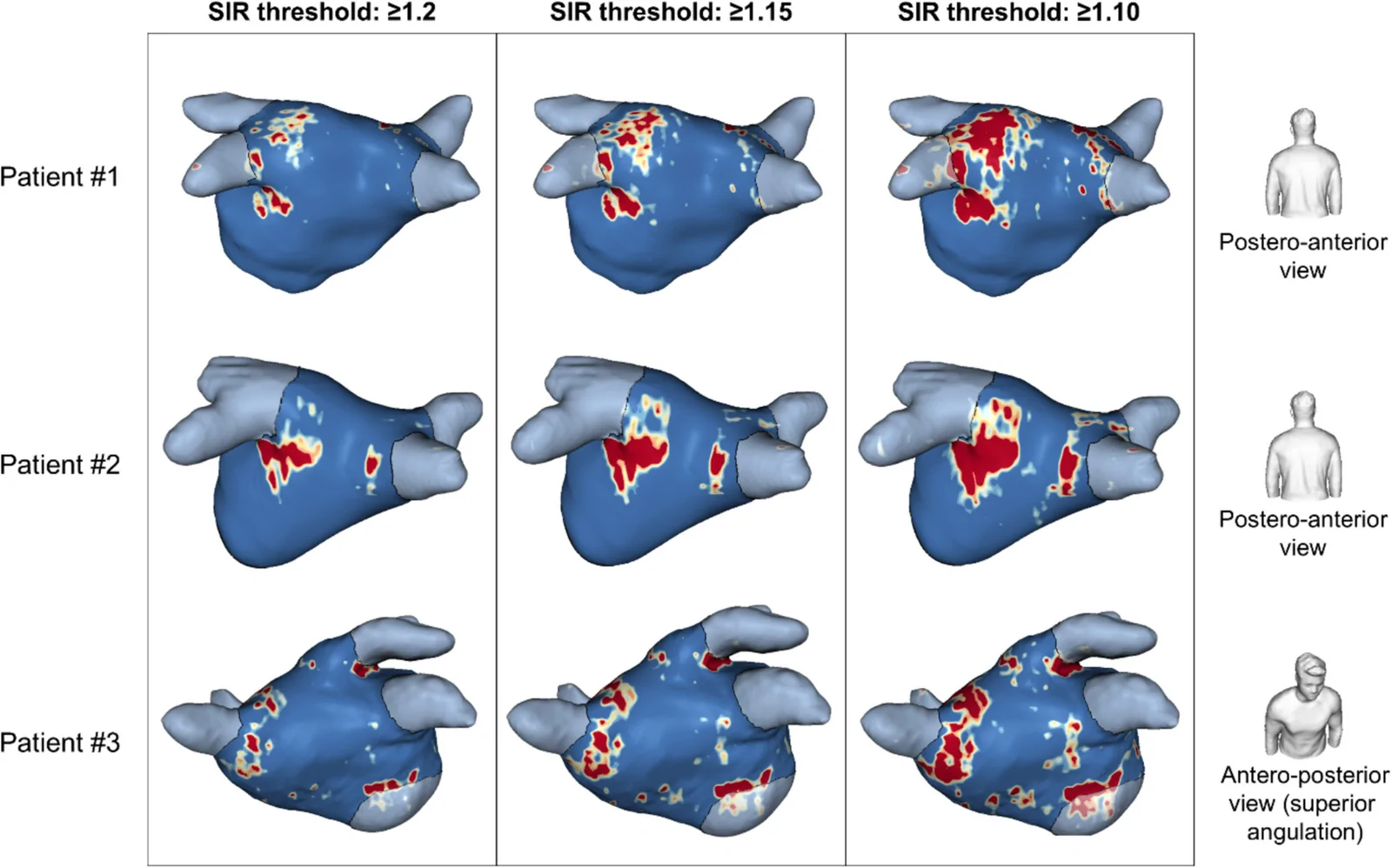
Representative PFA lesion sets according to different lesion-defining signal intensity ratio (SIR) thresholds. Left atrial 3D-reconstructions from three PFA patients, 3 months post-ablation (PVI-only), with color-coding based on LGE-defining signal intensity ratios. Left column: Scar (red, > 1.32; yellow, 1.2–1.32), normal tissue (blue, < 1.2). Middle column: Scar (red, > 1.27; yellow, 1.15–1.27), normal tissue (blue, < 1.15). Right column: Scar (red, > 1.22; yellow, 1.10–1.22), normal tissue (blue, < 1.10)

Given the relatively low continuity of ablation lesions detected after PFA, we repeated the analyses of post-PFA lesions using two lower LGE-defining thresholds to enhance lesion detection. As expected, application of decreased signal intensity ratio thresholds defining late gadolinium enhancement indicative of ablation lesions increased the sensitivity of lesion detection, which resulted in lower cumulative gap lengths (SIR ≥ 1.2, 36%; ≥ 1.15, 29%; SIR ≥ 1.10, 23%; *p* < 0.01) and a higher proportion of patients with complete lesions in the PFA group (SIR ≥ 1.2, 12%; ≥ 1.15, 20%; SIR ≥ 1.10, 25%; *p* = 0.35). However, based on visual assessment, we must assume that the increased sensitivity achieved with lower lesion–defining thresholds is at the cost of a relevant decrease in specificity (see Fig. 5 for representative images).

Of note, lesion continuity was generally higher at the left-sided than at the right-sided PVs as indicated by lower cumulative gap lengths. This difference between left and right PVs equally applied to all ablation techniques (Table 2).

**Table 2:** Lesion characteristics in to left- versus right-sided PVs

	Cumulative gap length (%)			Lesion width (mm)		
	LPV	RPV	*p*-value	LPV	RPV	*p*-value
**RF**	12.9 (6.8–19.0)	24.8 (18.7–30.9)	0.0001	9.2 (8.3–10.1)	8.2 (7.3–9.1)	0.0181
**CRYO**	9.4 (3.1–15.8)	27.1 (20.8–33.5)	< 0.0001	14.2 (13.3–15.1)	12.2 (11.2–13.1)	< 0.0001
**HPSD**	11.3 (3.2–19.3)	18.6 (10.5–26.6)	0.0630	12.3 (11.2–13.5)	9.3 (8.1–10.49)	< 0.0001
**PFA**	19.8 (12.4–27.2)	51.2 (43.8–58.7)	< 0.0001	13.9 (12.8–15.0)	11.8 (10.6–13.1)	0.0004

All values are estimated means with the corresponding 95% confidence interval. *LPV*, left pulmonary vein; *RPV*, right pulmonary vein

### Ablation lesion geometry

**Fig. 6:**
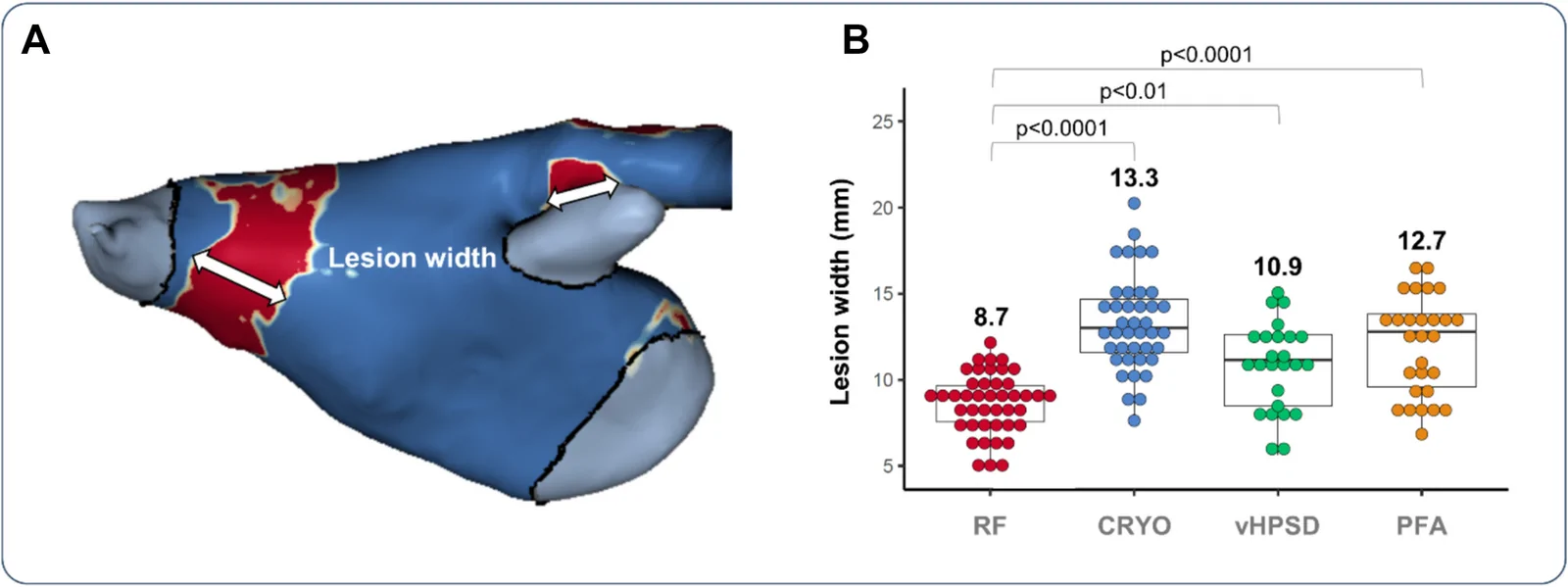
Average lesion width. **A** Representative 3D reconstruction visualizing post-ablation LGE lesions based on signal intensity ratios with distinct thresholds for scar (red, > 1.32; yellow, 1.2–1.32) and normal tissue (blue, < 1.2) (ADAS 3D). Arrows indicate lesion width measurements. **B** Mean lesion widths. Data are presented as box plots with individual data points. Only *p*-values < 0.05 are displayed

To compare ablation lesion geometries, the mean lesion width was assessed. PV-encircling LGE lesions were widest in the cryoballoon (mean lesion width 13.3 mm) and the PFA group (12.7 mm) (Fig. 6). Interestingly, HPSD ablation lesions were significantly wider than conventional RF ablation lesions (mean lesion width 10.9 vs. 8.7 mm, *p* = 0.0083). Of note, LGE lesions were generally wider at the left-sided compared to the right-sided PVs (Table 2). This equally applied to all ablation techniques.

### Clinical outcome

Although this study was neither designed nor powered for clinical endpoints, a systematic follow-up was performed to assess arrhythmia recurrence. There was no significant difference in 12 months arrhythmia-free survival between the different groups (HPSD 84%; conventional RF 70%; CRYO 70%, PFA 80%; Log rank test: *p* = 0.451).

## Discussion

This is the first controlled study to systematically compare ablation lesions from novel approaches like PFA and HPSD ablation with conventional approaches like ablation index–guided RF ablation or latest generation cryoballoon ablation. Our head-to-head comparison of all four ablation approaches revealed considerable differences in lesion continuity and geometry.

Interestingly, HPSD ablation resulted in the most continuous lesions and the highest proportion of complete PV-encircling LGE lesions without any gaps, whereas PFA lesions displayed the most and largest discontinuities. As expected, large-area ablation with the cryoballoon and PFA single-shot devices resulted in the widest lesions. However, it is noteworthy that HPSD lesions were significantly wider than conventional RF lesions.

There was a consistent trend of more pronounced lesion formation in left versus right PVs throughout the different ablation techniques. However, we are unable to distinguish whether this observation is due to more effective ablation or more sensitive detection of lesions by MRI in left versus right PVs. Anyway, a similar trend has been reported previously, and it may warrant further investigation [31].

### High-power short-duration ablation

The fact that HPSD ablation resulted in the most continuous lesions is in line with preclinical data [14, 42]. In fact, this could be a result of a wider and shallower lesion geometry as postulated based on experimental studies, and indeed, our finding of a higher width of HPSD compared to conventional RF ablation lesions further corroborates this hypothesis [14, 42].

### PFA

While PFA-induced LGE lesions cover relatively large areas, comparable to cryoballoon ablation, the LGE distribution is far more inhomogeneous. The discontinuous lesions resulting from PFA are suggestive of a low PVI durability, and indeed, available data are somewhat inconsistent in this respect: Systematic invasive remapping 3 months post-PFA in the Farapulse approval trial showed high rates of durable PVI, with complete isolation of all PVs encountered in > 80 of the patients [8, 43]. On the other hand, recent studies reported relatively low proportions of durable complete PVI (21–42%) in patients undergoing redo procedures because of arrhythmia recurrence after PFA using the Farapulse system [13, 44–47]. Of note, the proportion of durable complete PVI encountered in patients with recurrent arrhythmias after ablation index–guided RF ablation following the CLOSE protocol was 62% in a study by De Pooter et al. [48]. As with thermal ablation techniques, tissue contact seems to be an important determinant of lesion quality and durability with PFA [49–52]. While special care was taken in our study to ensure adequate tissue contact by visual assessment of the backwards bending of the flexible catheter splines, a local lack of sufficient tissue contact cannot be fully ruled out.

Another aspect to be taken into account is the limited operator experience with the Farapulse PFA system. While operators were highly experienced with conventional RF ablation and cryoballoon ablation (minimum of 10 years experience with each of the two), the novelty of HPSD ablation with the QDOT catheter and the PFA approach inherently implies that they were less experienced with those techniques. Thus, we cannot rule out that the limited experience has negatively impacted the lesion quality accomplished with the novel techniques. However, for PFA using the Farapulse system, it is meanwhile well established that it has a very flat learning curve, particularly in operators that are highly experienced with single-shot devices like the cryoballoon [12]. On the other hand, it has to be emphasized that inhomogeneous PFA lesions might as well reflect a different kind of tissue remodeling in response to PFA compared to thermal ablation, which may be less detectable by LGE-CMR. Indeed, limitations in detectability by LGE-CMR have been suggested previously by Nakatani et al. [53]. Finally, both, limited lesion quality and limited lesion detectability, may explain the observed phenomenon of inhomogeneous PFA lesions to some extent.

If detectability of PFA lesions was a limiting factor indeed, this may be improved by lower signal intensity ratio thresholds defining LGE indicative of ablation lesions. Our data indicate that, as expected, lower thresholds increase sensitivity in the detection of PFA lesions, but that this is likely to be at the cost of a limited specificity. In fact, the standard ablation lesion-defining threshold used in our study (SIR ≥ 1.2) has been extensively validated in the past, to optimize sensitivity and specificity [31, 32, 38, 54]. Thus, while thresholds may have to be adapted for non-thermal ablation techniques like PFA, systematic and prospective validation of thresholds would be required.

### Clinical implications

#### Lesion continuity

This study was not designed to investigate clinical endpoints, and the sample size is sufficient to detect potential differences in AF- or arrhythmia-free survival with adequate power. However, we have extensively validated the clinical implications of LGE-CMR-based ablation lesion parameters in recent years. In fact, cumulative gap length has been linked to arrhythmia recurrence, and LGE lesions covering > 90% of the PV antral circumference were shown to be associated with very high rates of arrhythmia-free survival [38]. Moreover, complete PV-encircling LGE lesions without any gaps were found to rule out PV reconnection with a negative predictive value of 97% in patients undergoing redo procedures for recurrent AF [32].

#### Lesion size and potential iatrogenic substrate

Regarding the different extents of ablation lesions, it is tempting to speculate that wider, and thus larger area lesion, particularly if inhomogeneous, may constitute a potential substrate for persistent AF. To date, the concept of iatrogenic substrate has been limited to gaps in ablation lines or localized ablation lesions that enable post-ablation atrial tachycardias based on reentry mechanisms [55, 56]. However, diffuse fibrosis potentially resulting from inhomogeneous large-area ablations may harbor a more complex substrate promoting persistent AF—even in the absence PV-reconnecting gaps.

Intriguingly, a recent post-hoc analysis of the CIRCA-DOSE trial found PVI using RF ablation to be superior to cryoballoon ablation in the prevention of disease progression to persistent AF, despite similar recurrence rates and AF burden [3]. To date, an explanation for this observation has not been provided, but it might be related to the composition and extent (and thus arrhythmogeneicity) of the ablation lesions, which varies substantially between the different ablation approaches and energy sources.

#### Evidence from randomized trials

Finally, it remains unknown, to what extent the differences in lesion characteristics of novel ablation technologies observed in this study may impact clinical outcome. While the clinical efficacy of HPSD ablation using the QDOT catheter has not been assessed in a randomized trial so far, PFA using the Farapulse system was compared with conventional thermal ablation in the recent ADVENT randomized trial: At least in this study, which demonstrated non-inferiority of PFA, any putative inhomogeneity of PFA lesions did apparently not have a detectable impact on AF recurrence [9]. In line with these data, our study did not detect any difference in 12-months arrhythmia-free survival between the different groups, although it must be taken into account that the study was neither designed nor powered for clinical endpoints.

### Limitations

As a result of the non-randomized study design that does not prevent selection bias, patient characteristics are not evenly distributed, with a higher proportion of patients with paroxysmal AF in the CRYO and PFA groups. However, while this would probably impact clinical outcomes in terms of arrhythmia recurrence rates, an effect on lesion formation is unlikely.

As outlined above, with respect to the inhomogeneous PFA lesions, we cannot determine the relative contribution of inferior lesion quality on the hand and limited lesion detectability, since validation through systematic invasive remapping had not been included in the study protocol, due to ethical concerns and understandable reservations of the patients.

Finally, conclusions based on the QMODE HPSD ablation and the Farapulse PFA, respectively, may not necessarily apply to different HPSD or PFA approaches.

## Conclusions

It has to be acknowledged that ablation lesions differ fundamentally among different ablation concepts and technologies. Of the four concepts compared in this study, HPSD ablation resulted in the most continuous lesions. Of note, HPSD lesions were also significantly wider than conventional RF lesions, thus corroborating the concept of a shallower HPSD lesion geometry from experimental studies.

While PFA lesions cover relatively large areas, comparable to cryoballoon ablation, lesions are more inhomogeneous. However, it remains to be determined to what extent LGE discontinuities in PFA lesions indicate ineffective ablation or only reflect a different kind of remodeling compared to thermal ablation, which may be less detectable by LGE-MRI.

## Data Availability

The data underlying this article will be shared upon reasonable request to the corresponding author.

## Abbreviations

PV: Pulmonary vein
PVI: Pulmonary vein isolation
AF: Atrial fibrillation
PFA: Pulsed-field ablation
HPSD: High-power short-duration
LGE: Late gadolinium enhancement
CMR: Cardiac magnetic resonance
CRYO: Cryoballoon
RF: Radiofrequency
